# Carbon Concerns: Nanotubes Cause Cardiovascular Damage

**Published:** 2007-03

**Authors:** Ernie Hood

Lung deposition of single-wall carbon nanotubes (SWCNTs), one of the most commonly used materials in nanotechnology, is already known to cause localized toxic effects. Now scientists have demonstrated that such deposition also leads to cardiovascular damage in mice, including accelerated formation of atherosclerotic plaques **[*EHP* 115:377–382; Li et al.]**. The findings add to concerns that exposure to SWCNTs could result in systemic toxic effects.

The team conducted a series of experiments, instilling SWCNTs into the lungs of mice. In an initial screen for extrapulmonary effects, *Ho1luc* reporter transgenic mice were exposed to single SWCNT doses of 10 or 40 μg. Heme oxygenase-1 (HO-1) gene expression, a biomarker of oxidative stress, was activated in the animals’ lung, aorta, and heart tissue at 7 days post-exposure, declining to control levels by day 28. This held with pulmonary toxicity studies showing an early, transitory inflammatory response.

The same dosing scheme was used in experiments with the commonly used C57BL/6 mouse, which showed dose-dependent aortic mitochondrial DNA (mtDNA) damage at 7, 28, and 60 days post-exposure. mtDNA is highly susceptible to oxidative damage, considered to be an initiating event in atherogenesis. Among the treatment groups, glutathione and protein carbonyl levels—two other indicators of oxidative stress—were also significantly reduced and increased, respectively, adding to the evidence that exposure to SWCNTs can lead to oxidative insult. Exposure to comparable doses of ultra-fine carbon black particles in a control group produced no such damage to aortic mtDNA.

The group then tested the effects of SWCNT exposure in ApoE^−/−^ mice, a widely used model of human atherosclerosis. They exposed the mice to 20 μg of SWCNTs once every other week for 8 weeks. Then the mice were fed either a regular chow diet or a high-fat diet for the first half of that period to induce the elevated lipid concentrations that often precede atherosclerosis. Although SWCNT exposure was not associated with changes in the animals’ lipid profiles, the exposed mice on the high-fat regimen did exhibit accelerated plaque formation in the aorta and brachiocephalic arteries compared with controls.

The researchers note that the cardiovascular effects resulting from SWCNT exposure could be either direct, as a result of translocation of particles from the lung into the systemic circulation, or indirect, caused by the release of inflammatory mediators in the lung or by altered pulmonary function (although no increase in several measured inflammatory mediators was detected in the exposed animals). Whichever mechanism may be at work, these data show that lung deposition of SWCNTs, a possible workplace exposure scenario, can cause systemic damage and may contribute to cardiovascular disease.

## Figures and Tables

**Figure:**
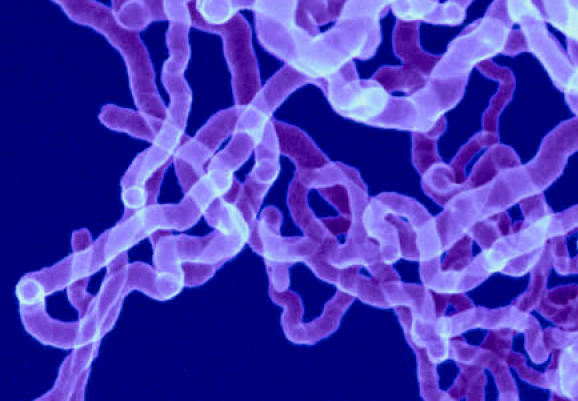
Worming their way in? SWCNTs may cause systemic toxicity.

